# Effect of smoking status on total energy expenditure

**DOI:** 10.1186/1743-7075-7-81

**Published:** 2010-11-01

**Authors:** David P Bradley, Lindsey A Johnson, Zhumin Zhang, Amy F Subar, Richard P Troiano, Arthur Schatzkin, Dale A Schoeller

**Affiliations:** 1 Department of Endocrinology, School of Medicine and Public Health, University of Wisconsin, Madison, WI, USA; 2 Department of Nutritional Sciences, University of Wisconsin, Madison, WI, USA; 3 Catering and Conference Service, University of Wisconsin, Madison, WI, USA; 4 Division of Cancer Epidemiology and Genetics, National Cancer Institute, National Institutes of Health, Department of Health and Human Services, Rockville, MD, USA; 5 Division of Cancer Control and Population Sciences, National Cancer Institute, National Institutes of Health, Bethesda, MD, USA

## Abstract

Individuals who smoke generally have a lower body mass index (BMI) than nonsmokers. The relative roles of energy expenditure and energy intake in maintaining the lower BMI, however, remain controversial. We tested the hypothesis that current smokers have higher total energy expenditure than never smokers in 308 adults aged 40-69 years old of which 47 were current smokers. Energy expenditure was measured by doubly labeled water during a two week period in which the subjects lived at home and performed their normal activities. Smoking status was determined by questionnaire. There were no significant differences in mean BMI (mean ± SD) between smokers and never smokers for either males (27.8+5.1 kg/m^2 ^vs. 27.5+4.0 kg/m^2^) or females (26.5+5.3 kg/m^2 ^vs. 28.1+6.6 kg/m^2^), although the difference in females was of similar magnitude to previous reports. Similarly, total energy expenditure of male smokers (3069+764 kcal/d) was not significantly different from that of never smokers (2854+468 kcal/d), and that of female smokers (2266+387 kcal/d) was not different from that of never smokers (2330+415 kcal/d). These findings did not change after adjustment for age, fat-free mass and self-reported physical activity. Using doubly labeled water, we found no evidence of increased energy expenditure among smokers, however, it should be noted that BMI differences in this cohort also did not differ by smoking status.

## Introduction

Numerous cross-sectional studies have indicated that body mass index (BMI) is lower in cigarette smokers than in nonsmokers [1-3], and that leanness correlated directly with duration, but not intensity of smoking, with longer duration associated with lower BMI [4-6]. Recent statistical analyses of data sets from both the 2005-2006 National Health and Nutrition Examination Survey (NHANES) [2] and the 2005 National Health Interview Survey [7] confirmed findings from prior studies indicating that smokers weighed significantly less than nonsmokers. Individuals who stop smoking have been reported to gain weight after they quit, and this prospect can discourage tobacco cessation [1,3,6]. Women seem to be somewhat more susceptible to weight gain following smoking cessation than men. In a 10 year study, the mean weight gain attributable to cessation was 5.0 kg in women and 4.4 kg in men [3]. Multiple studies have shown that 33-75% of ex-smokers reported weight gain within the first year of cessation [8].

While the exact cause of this weight gain is unclear, two hypotheses, which are not mutually exclusive, are frequently proposed. The first is that smoking increases energy expenditure due to nicotine's effects in raising metabolic rate, resulting in decreased energy expenditure with cessation [8-10]. In 1986, Hofstetter et al. reported that 24-hour energy expenditure increased in smokers by 140-200 kcal/day on a day with smoking compared to a day without smoking, and no corresponding change in mean basal metabolic rate [11]. Further studies have since yielded conflicting results with most finding an increase in resting metabolic rate (RMR) shortly after nicotine administration. Walker et al. found a 6% increase in RMR after 20 minutes of smoking [12] and Dalloso et al. documented a 3% increase in RMR after smoking a single cigarette [10]. Several studies, however, failed to find a corresponding increase in RMR measured either before or after smoking a single cigarette before they ceased smoking [13,14].

The second hypothesis is that smoking alters energy intake by inducing an anorexic effect [9] and, by extension, smoking cessation leads to weight gain due to increased intake [15]. Findings on this subject vary and results are also inconsistent. In a study by Jessen et al., during a two hour period, nicotine administration was negatively associated with hunger and food consumption and positively associated with satiety [16]. Perkins et al. found increased satiety and lower caloric intakes following nicotine administration [17]. However, in another study, the same authors found higher caloric intakes with nicotine administration [18].

Unfortunately, the second hypothesis is difficult to test because self-report tools were used to measure intake. Numerous studies have shown that self-reported dietary records are prone to error, usually in the direction of underreporting [19]. In addition, the first hypothesis has historically been difficult to test, with the exception of short-term measurements of RMR over a period of minutes or hours. This is because measuring total energy expenditure (TEE) under free-living conditions was difficult in the past [20]. This has changed, however, with the development of the doubly labeled water (DLW) method for measuring TEE [21]. This method is based on the observation that after a loading dose of water labeled with deuterium and ^18^O, the deuterium is eliminated from the body as water, while the ^18^O is eliminated as water and carbon dioxide. The difference between the elimination rates is therefore proportional to carbon dioxide production and hence energy expenditure [21]. The doubly labeled water technique provides a measure of TEE which includes RMR, thermic effects of meals (TEM) and physical activity energy expenditure (PAEE). Moreover, the DLW method averages TEE over a period of days to two weeks thus providing a counterpart to energy intake in assessing contributions to energy balance [21].

Utilizing DLW, Warwick and Baines reported no difference in energy expenditure between smokers and non-smokers. This study, however, included only 11 smokers and 10 nonsmokers and thus had limited power [22]. We calculate the standard error for the difference in TEE between smokers and nonsmokers in the study of Warwick and Barns to be 249 kcal/d and thus the smallest difference they could have expected to identify (P < 0.05) with 80% power would have been nearly 700 kcal/d.

We analyzed data from the National Cancer Institute's (NCI) Observing Protein and Energy Nutrition (OPEN) Study, which was conducted to assess the structure of measurement error in self-reported dietary assessment instruments. In OPEN, TEE was measured by DLW in 484 adults [23,24]. Each participant also reported their smoking status, thus providing an opportunity to test the hypothesis that the TEE of smokers is greater than that of never-smokers.

## Methods

### Subjects

Of the original 484 adults (age 40-69 years) who participated in the OPEN study, which was conducted during 1999, 10 were excluded because they lacked smoking data, 150 were excluded because they had smoked at one time, but were currently non-smokers, and 33 were excluded because of missing energy expenditure data [23]. The remaining 304 participants (158 male, 146 female), 47 of which (20 male, 27 female) were current smokers were included in the analysis. The majority of the participants in OPEN were white males and females with some college education or higher [25]. The study was approved by the institutions involved and participants provided signed informed consent.

### Study Design

The OPEN Study evaluated the structure of dietary measurement error in food frequency questionnaires (FFQs) and 24-hour dietary recalls (24 HRs) by using DLW and urinary nitrogen as biomarkers of TEE and protein. Full details can be found elsewhere, including dietary data [25,26]. In brief, a randomized sample of 5,000 individuals aged 40-69 years of age within the Washington DC area was contacted via telephone. After an initial telephone encounter, participants meeting inclusion criteria were invited to a series of 3 clinic visits. DLW studies were performed between the first visit and second visit (11-14 days later).

DLW studies were performed using a five-urine-specimen protocol. DLW was administered orally at the first visit at a dose of 2 g of 10 atom percent ^18^O labeled water and 0.12 g of 99.9 atom percent ^2^H labeled water per kilogram of estimated total body water along with a 50-ml rinse of the dose bottle. Urine specimens were then collected at 2, 3, and 4 hours after the dose with the 2 hour specimen being discarded. Urine was again collected twice approximately 14 days later. TEE was calculated with the modified Weir equation, assuming a respiratory quotient of 0.86. Isotope analysis was performed as previously described [25].

Participants reported their smoking status and physical activity level on questionnaires. Smoking status was based on the number of cigarettes smoked during the lifetime. Those who had smoked less than 100 cigarettes in their lifetime were classified as never-smokers and those who had smoked more than 100 cigarettes were classified as smokers. Those who reported quitting smoking were classified as past smokers and those who did not report quitting were classified as current smokers. Only never smokers and current smokers were included in the study. The DLW method thus provided a measure of total daily energy expenditure averaged over a period 11 to 14 days during which there were no restrictions or instructions regarding smoking.

Physical activity was assessed with the OPEN Study Physical Activity Questionnaire, the same questionnaire that was used in NHANES 2001-2002 [27]. Participants reported the duration and frequency of specific domains of physical activity, which were translated into MET-minutes of physical activity per week.

The FFQ utilized in the OPEN study was the Diet History Questionnaire (DHQ) (available at http://www.riskfactor.cancer.gov/DHQ/), developed and evaluated at NCI [25]. This questionnaire assessed the frequency of intake for 124 individual foods/food group items during the preceding 12 months and evaluated the portion size of most items. Data from the DHQ was analyzed by Diet*Calc Software (version 1.4.3, 2005, National Cancer Institute, Bethesda, MD).

The 24 HR used in the OPEN Study was a standardized five-pass method, developed by the US Department of Agriculture for use in national dietary surveillance [26].

Fat free mass (FFM) was calculated indirectly based on TBW from the deuterium and O^18 ^dilution spaces and body mass.

### Statistical Analysis

All statistical analyses were performed using SAS (version 9.13, SAS Institute, Inc, Cary NC). Male and female participants were analyzed separately using a T-test to assess smoking-group differences for continuous outcomes. Multiple regression models were fitted to compare the TEE of current smokers to never smokers including four variables significantly influencing TEE: gender, age (years), fat free mass (FFM) (kg), and physical activity (PA) (MET minutes/week). Least-squares (LS) means were also calculated and compared between smokers and non-smokers. All regression analyses were performed using the PROC GLM procedure in SAS. An α value of < 0.05 was considered statistically significant.

## Results

Subject characteristics are shown in Table 1. Among both males and females, no significant differences were detected between smokers and non-smokers for age, height, weight, BMI, FFM, or reported PA. Consistent with other studies, the female participants, on average, expended less energy than the males (2319 +/- 409 kcal/day vs. 2882 +/- 518 kcal/day, p < 0.001, Table 2). When categorized by smoking status, unadjusted TEE in female smokers and never smokers averaged 2266 ± 387 kcal/day and 2330 ± 415 kcal/day respectively and was not significantly different (p = 0.46). Among males, unadjusted average TEE was 3069+764 kcal/d in smokers and 2854+468 kcal/d in never smokers, also not significantly different (p = 0.08).

**Table 1:** Subject Characteristics

	Males		Females	
	Smokers (n = 20)	Never-Smokers (n = 138)	Smokers (n = 27)	Never-Smokers (n = 119)
Age (y)	50.6 ± 8.7	53.4 ± 8.3	53.6 ± 8.1	52.2 ± 7.9
Height (cm)	177.4 ± 7.7	176.3 ± 7.9	164.6 ± 6.6	162.3 ± 6.7
Weight (kg)	87.7 ± 18.9	85.4 ± 13.9	71.8 ± 16	74.1 ± 18.3
BMI (kg/m^2^)	27.8 ± 5.1	27.5 ± 4.0	26.5 ± 5.3	28.1 ± 6.6
FFM (kg)	60 ± 11.4	58 ± 7.9	42.8 ± 7.1	42.4 ± 6.7
				
Physical Activity (MET min/wk)	1128 ± 1154	1558 ± 1411	1467 ± 1679	1516 ± 1387

Values are written as mean ± standard deviation. FFM = Fat Free Mass.

**Table 2:** TEE in smokers and non-smokers

	Overall	Never smokers	Current smokers	Difference
Males	2882 ± 518	2854 ± 468	3069 ± 764	214 (p = 0.08)
Females	2319 ± 409	2330 ± 415	2266 ± 387	64 (p = 0.46)

Values are written as mean +/- standard deviation in kcal/day. The number of subjects is the same as in Table 1.

FFM and age were significantly correlated with TEE in both genders (Table 3). The effect of smoking status on TEE is shown in Table 4. No difference in least-square means was found.

**Table 3:** Pearson's correlation between TEE and related factors

Factor	Correlation coefficient (r)	p-value
**Females**		
FFM (kg)	0.75	p < 0.001
Age (y)	-0.34	p < 0.001
SR PA (MET*h)	0.13	p = 0.13
**Males**		
FFM (kg)	0.76	p < 0.001
Age (y)	-0.21	p = 0.001
SR PA (MET*h)	0.12	p = 0.16

The number of subjects is the same as in Table 1. SR PA = Self reported physical activity in mets/hour.

**Table 4:** Comparison of total energy expenditure (kcal/d) by smoking status in males and females after controlling for the effects of age, fat-free mass, and self-reported physical activity.

	Never smokers	Current Smokers	Difference
**Males**			
Adjusted for age and FFM	2870+28	2961+72	91 (p = 0.24)
Adjusted age, FFM, and SR PA	2867+27	2979+71	111 (p = 0.15)
**Females**			
Adjusted age and FFM	2334+24	2252+51	-81 (p = 0.16)
Adjusted for age, FFM, and SR PA	2334+24	2252+51	-81 (p = 0.15)

Values are given as least square mean +/- standard error of the mean. Age in years, FFM = fat-free mass in kg, and SR PA is self reported physical activity in mets*hour. The number of subjects is the same as in table 1.

Table 5 shows the multiple regression coefficients for several factors related to TEE. Smoking was not significantly associated with TEE in either males or females. FFM was the strongest factor positively associated with TEE in both males and females, while age and PA were positively associated in males, but not in females.

**Table 5:** Multiple Regression Coefficients for Factors Related to Total Energy Expenditure

	Coefficient estimation	p-value
**Males**		
Smoking	111.9	p = 0.15
FFM	43.6	p < 0.001
Age	-13.3	p < 0.001
SR PA	0.05	p = 0.006
**Females**		
Smoking	-81.8	p = 0.15
FFM	45.0	p < 0.001
Age	-3.5	p = 0.21
SR PA	0.01	p = 0.40

FFEM = Fat free mass. SR PA = Self reported physical activity.

## Discussion

This is the largest study to compare total energy expenditure between free-living smokers and never-smokers using DLW. In so doing, we greatly strengthen the findings of Warwick and Baines, who also reported no difference in energy expenditure between smokers and non-smokers (2877+/- 496 kcal/d vs. 2541 +/- 628 kcal/d), by including 47 smokers and 257 non-smokers compared to their small sample of 11 and 10 smokers and non-smokers [22]. Our larger sample, therefore, allowed us to detect a difference of 216 kcal/d compared with 700 kcal/d for the smaller study. Based on the unadjusted TEE value, the 95% CL for the TEE difference between male smokers and never smokers was -28 to 458 kcal/d and among females the 95% CL of the difference was -237 to 109 kcal/d. TEE, however, was strongly correlated with FFM and thus should be adjusted for FFM as well as age [23]. After adjustment, the 95% CL for TEE in male and female smokers compared to never smokers was -64 to 246 kcal/d and -193 to 29 kcal/d, respectively.

Other investigators have compared the energy requirements of smokers and non-smokers using dietary records with mixed results [28-35]. Such conflicting findings are not surprising in light of data showing that many individuals under-report their dietary energy intake by amounts often in excess of 15% indicating that the use of self-reported energy intake as a proxy measure of energy expenditure can be problematic [24,36]. In contrast, the DLW method has been carefully validated against measured TEE in a metabolic chamber and shown to be accurate within 1-2% with a coefficient of variation of 4-7% [37].

Our finding of no significant difference in the TEE between current smokers and never smokers appears inconsistent with previous studies indicating that the RMR component of TEE is higher in smokers than non-smokers. For example, following multiple administrations of two nicotine doses to eighteen male smokers, Perkins et al. found that RMR acutely increases 3% above placebo after both moderate and low doses [38]. Likewise, Dallosso and James found that metabolic levels were elevated by 2.8% and 1.5%, 1-30 and 31-60 min after smoking [10]. This increase appears to be acute rather than long-term. No animal studies have found a significant increase in long-term RMR after frequent exposure to cigarette smoke or nicotine [39,40]. Similarly, most human studies found that smoking has insignificant long-term effects on RMR [13,14,40].

Unfortunately, RMR was not measured in the OPEN Study and thus we cannot direct compare results with regard to RMR. We can, however, calculate the effect of a short-term post-smoking percentage change in RMR to TEE. Assuming that smoking causes a 3 to 5% increase in RMR for up to 1 h after smoking, we would predict that a person who smokes a pack a day of cigarettes would have an increase in TEE of 30 to 50 kcal/day. This small change is inside the confidence interval for our results in males and too small to detect. In females, however, we can reject the hypothesis that smoking causes a 30-50 kcal/d higher TEE in females. It should be noted, however, that our results are not necessarily inconsistent with a small increase in RMR. If one assumes that smoking is primarily a sedentary activity, then it is possible that the time spent smoking leads to reductions in physical activity that may compensate any potential increase in RMR associated with smoking. To test for this possibility, we also adjusted TEE for self-reported physical activity, but that adjustment had no influence on the results. Self-reported physical activity, however, is also subject to reporting error and thus our conclusion regarding this hypothesis is weak [41].

One limitation of the DLW technique is that it measures CO_2 _production and inhaled cigarette smoke contains CO_2 _produced from the burning of the tobacco [38]. Thus CO_2 _that is absorbed by the body will result in an inherent overestimation of energy expenditure in the DLW based measurement. Assuming that the consumed portion of each cigarette contains 0.7 g of combustible material and that the smoker inhales two-thirds of the CO_2 _produced by the cigarette, smoking two packs a day would produce an error of about 0.6 mol of CO_2 _per day or 2-4% of true CO_2 _production. Thus, heavy smoking is expected to result in an overestimate of CO_2 _production and therefore energy expenditure [38]. This limitation of the DLW method, however, does not alter our conclusion that smoking does not increase energy expenditure compared to never smokers, as correction for inhaled CO_2 _would act to further reduce the TEE of our smokers by 45 to 115 kcal/day.

A second limitation of this analysis is that the OPEN cohort is not a representative sample. In particular, the male participants, who were classified as current smokers, did not have numerically lower BMIs than nonsmokers. This atypical BMI pattern may be due to behavioral differences between OPEN participants and that of other studies as the OPEN cohort was highly educated, (83% with one year of college or more including 32% with post-graduate education), from the same geographic area (Bethesda, MD and surrounding communities) and likely had a high level of health consciousness related to volunteer bias. Thus it is possible that the OPEN current smokers may have smoked less than participants in other studies resulting in the atypical BMI pattern seen in males

While it is true that dietary reporting may be associated with under eating [42], this is only true for food records as subjects know they are being observed. The OPEN study, however, utilized FFQs and 24-hour dietary recalls. FFQs ask about intake the past year while recalls are unannounced and ask about intake "yesterday." Both may be subject to error and bias but not to under eating. Under eating, if it did exist, would have an effect on caloric intake but very minimal effect on total energy expenditure which is the endpoint of our study.

Our findings among female participants, however, are more typical in that the BMIs of current smokers were 1.6 kg/m^2 ^less than those of never smokers. The adjusted TEE was not only not greater than that of never smokers, but actually trended toward a lower value than those of never smokers (p = 0.15) and thus did not support a hypothesis that TEE is elevated in smokers.

Our TEE findings among the participants from the OPEN study do not support the hypothesis that smoking increases TEE. These findings differ from those found in NHANES in which clear differences in BMIs between smokers and nonsmokers were shown. Also, although this is the largest reported cohort of smokers who have TEE data as measured by the DLW method, the total number of current smokers was still only 47 individuals, thus power was limited.

These indicate that weight gain following smoking cessation is a result of increased caloric consumption versus decreased TEE, indicating that health practitioners should include dietary counseling in smoking cessation programs with an eye toward decreasing post-cessation weight gain.

## Competing interests

The authors declare that they have no competing interests.

## Authors' contributions

DPB drafted the manuscript. RPT, AFS and AS were involved in the conception of the study, conception and conduct of the OPEN Study, data interpretation, and manuscript preparation; DAS was involved in the conception of the study, analysis of doubly labeled water data, data interpretation, and manuscript preparation. ZZ and LAJ were involved in analysis of doubly labeled water data and data interpretation. All authors read and approved the final manuscript.

## References

[B1] Williamson DF Madans J Anda RF Kleinman JC Giovino GA Byers T Smoking cessation and severity of weight gain in a national cohort N Engl J Med 1991 324 739 45 10.1056/NEJM199103143241106 1997840

[B2] LaRowe TL Piper ME Schlam TR Fiore MC Baker TB Obesity and Smoking: Comparing Cessation Treatment Seekers with the General Smoking Population Obesity 2009 17 6 1301 1305 19247276 10.1038/oby.2009.36 PMC2918403

[B3] Flegal KM Troiano RP Pamuk ER Kuczmarski RJ Campbell SM The influence of smoking cessation on the prevalence of overweight in the United States N Engl J Med 1995 333 18 1165 70 10.1056/NEJM199511023331801 7565970

[B4] Albanes D Jones DY Micozzi MS Mattson ME Associations between smoking and body weight in the U.S. population: analysis of NHANES II Am J Public Health 1987 77 4 439 44 10.2105/AJPH.77.4.439 3493709 PMC1646954

[B5] Ward KD Klesges RC Vander Weg MW Cessation of smoking and body weight International textbook of obesity 2001 Björntop P Chichester, United Kingdom: Wiley & Sons Ltd 323 36 full_text

[B6] Klesges RC Winders SE Meyers AW Eck LH Ward KD Hultquist CM Ray JW Shadish WR How much weight gain occurs following smoking cessation? A comparison of weight gain using both continuous and point prevalence abstinence J Consult Clin Psychol 1997 65 2 286 91 10.1037/0022-006X.65.2.286 9086692

[B7] Kruger J Ham SA Prohaska TR Behavioral risk factors associated with overweight and obesity among older adults: the 2005 National Health Interview Survey Prev Chronic Dis 2009 6 1 19080020 PMC2644612

[B8] Parsons AC Shraim M Inglis J Aveyard P Hajek P Interventions for preventing weight gain after smoking cessation Cochrane Database Syst Rev 2009 1 19160269 10.1002/14651858.CD006219.pub2

[B9] Chiolero A Faeh D Paccaud F Cornuz J Consequences of smoking for body weight, body fat distribution, and insulin resistance Am J Clin Nutr 2008 87 4 801 9 Review 18400700 10.1093/ajcn/87.4.801

[B10] Dallosso HM James WP The role of smoking in the regulation of energy balance Int J Obes 1984 8 4 365 75 6511171

[B11] Hofstetter A Schutz Y Jequier E Wahren J Increased 24-hour energy expenditure in cigarette smokers N Engl J Med 1986 314 2 79 82 10.1056/NEJM198601093140204 3941694

[B12] Walker J Collins LC Nannini L Stamford BA Potentiating effects of cigarette smoking and moderate exercise on the thermic effect of a meal Int J Obes Relat Metab Disord 1992 16 5 341 7 1319968

[B13] Stamford BA Matter S Fell RD Papanek P Effects of smoking cessation on weight gain, metabolic rate, caloric consumption, and blood lipids Am J Clin Nutr 1986 43 4 486 94 3962901 10.1093/ajcn/43.4.486

[B14] Burse RL Bynum GD Pandolf KB Goldman RF Sims EAH Danforth E Increased appetite and unchanged metabolism upon cessation of smoking with diet held constant Physiologist 1975 18 157

[B15] Moffatt RJ Owens SG Cessation from cigarette smoking: changes in body weight, body composition, resting metabolism, and energy consumption Metabolism 1991 40 5 465 70 10.1016/0026-0495(91)90225-L 2023532

[B16] Jessen A Buemann B Toubro S Skovgaard IM Astrup A The appetite-suppressant effect of nicotine is enhanced by caffeine Diabetes Obes Metab 2005 7 4 327 33 10.1111/j.1463-1326.2004.00389.x 15955118

[B17] Perkins KA Epstein LH Stiller RL Fernstrom MH Sexton JE Jacob RG Solberg R Acute effects of nicotine on hunger and caloric intake in smokers and nonsmokers Psychopharmacology 1991 103 1 103 9 10.1007/BF02244083 2006236

[B18] Perkins KA Epstein LH Sexton JE Solberg-Kassel R Stiller RL Jacob RG Effects of nicotine on hunger and eating in male and female smokers Psychopharmacology 1992 106 1 53 9 10.1007/BF02253588 1738793

[B19] Trabulsi J Schoeller DA Evaluation of dietary assessment instruments against doubly labeled water, a biomarker of habitual energy intake Am J Physiol Endocrinol Metab 2001 281 E891 E899 11595643 10.1152/ajpendo.2001.281.5.E891

[B20] Haggarty P McGaw BA Non-restrictive methods for measuring energy expenditure Proceedings of the Nutrition Society 1988 41 365 374 10.1079/pns19880055 3151130

[B21] Schoeller DA van Santen E Measurement of energy expenditure in humans by doubly labeled water method J Appl Physiol 1982 53 4 955 9 6759491 10.1152/jappl.1982.53.4.955

[B22] Warwick PM Baines J Energy expenditure in free-living smokers and nonsmokers: comparison between factorial, intake-balance, and doubly labeled water measures Am J Clin Nutr 1996 63 1 15 21 8604664 10.1093/ajcn/63.1.15

[B23] Trumbo P Schlicker S Yates AA Poos M Dietary reference intakes for energy, carbohydrate, fiber, fat, fatty acids, cholesterol, protein and amino acids. Food and Nutrition Board of the Institute of Medicine, The National Academies J Am Diet Assoc 2002 102 11 1621 30 10.1016/S0002-8223(02)90346-9 12449285

[B24] Kipnis V Subar AF Midthune D Freedman LS Ballard-Barbash R Troiano RP Bingham S Schoeller DA Schatzkin A Carroll RJ Structure of dietary measurement error: results of the OPEN biomarker study Am J Epidemiol 2003 158 1 14 21 discussion 22-6 10.1093/aje/kwg091 12835281

[B25] Tooze JA Schoeller DA Subar AF Kipnis V Schatzkin A Troiano RP Total daily energy expenditure among middle-aged men and women: the OPEN Study Am J Clin Nutr 2007 86 2 382 7 17684209 10.1093/ajcn/86.2.382

[B26] Subar AF Kipnis V Trojano R Midthune D Schoeller DA Bingham S Sharbaugh CO Trabulsi J Runswick S Ballard-Barbash R Sunshine J Schatzkin A Using intake biomarkers to evaluate to extent of dietary misreporting in a large sample of adults: the OPEN study American Journal of Epidemiology 2003 158 1 13 10.1093/aje/kwg092 12835280

[B27] Physical activity questionnaire from the 1999-2000 National Health and Nutrition Examination Survey http://www.cdc.gov/nchs/data/nhanes/spq-pa.pdf Accessed September 20, 2010

[B28] Perkins KA Effects of tobacco smoke on caloric intake British Journal of Addiction 1992 87 2 193 205 10.1111/j.1360-0443.1992.tb02693.x 1554996

[B29] Margetts BM Jackson AA Interactions between people's diet and their smoking habits: the dietary and nutritional survey of British adults BMJ 1994 308 6935 1042 3 10.1136/bmj.307.6916.1381 PMC1679610 8274889

[B30] Cade J Margetts B Cigarette smoking and serum lipid and lipoprotein concentration British Medical Journal 298 1312 10.1136/bmj.298.6683.1312-a 2500214 PMC1836515

[B31] Fehily A Phillips K Yarnell J Diet, smoking, social class, and body mass index in the Caerphilly Heart Disease Study American Journal of Clinical Nutrition 1984 40 827 833 6486090 10.1093/ajcn/40.4.827

[B32] Haste FM Brooke OG Anderson HR Bland JM Shaw A Griffin J Peacock JL Nutrient intakes during pregnancy; observations on the influence of smoking and social class American Journal of Clinical Nutrition 1990 51 29 36 2296927 10.1093/ajcn/51.1.29

[B33] Jacobs DR Gottenborg S Smoking and Weight: the Minnesota Lipid Research Clinic American Journal of Public Health 1981 71 391 396 10.2105/AJPH.71.4.391 7468879 PMC1619681

[B34] Klesges RC Eck LH Isbell TR Fulliton W Hanson CL The effects of smoking status on the dietary intake, physical activity, and body fat of adult men American Journal of Clinical Nutrition 1990 51 784 89 2333836 10.1093/ajcn/51.5.784

[B35] Triosi RJ Heinhold JW Vokonas PS Weiss ST Cigarette smoking, dietary intake, and physical activity: effects on body fat distribution- The Normative Aging Study American Journal of Clinical Nutrition 1991 53 1104 11 1850574 10.1093/ajcn/53.5.1104

[B36] Westerterp KR Goris AH Validity of the assessment of dietary intake: problems of misreporting Curr Opin Clin Nutr Metab Care 2002 5 5 489 93 10.1097/00075197-200209000-00006 12172471

[B37] Schoeller DA Measurement of energy expenditure in free-living humans by using doubly labeled water J Nutr 1988 118 11 1278 89 3142975 10.1093/jn/118.11.1278

[B38] Perkins KA Epstein LH Stiller RL Marks BL Jacob RG Acute effects of nicotine on resting metabolic rate in cigarette smokers Am J Clin Nutr 1989 50 3 545 50 2773833 10.1093/ajcn/50.3.545

[B39] Wager-Sadr SA Levine AS Morley JE Hoidal JR Niewoehner DE Effects of Cigarette Smoke and Nicotine on Feeding and Energy Pharmacol. Biochem. Behav 1984 32 389 95 10.1016/0031-9384(84)90252-x 6463126

[B40] Perkins KA Metabolic Effects of Cigarette Smoking Journal of Applied Physiology 1992 72 2 401 9 1559911 10.1152/jappl.1992.72.2.401

[B41] Westerterp KR Assessment of physical activity: a critical appraisal Eur J Appl Physiol 2009 105 823 8 10.1007/s00421-009-1000-2 19205725

[B42] Goris A Westerterp-Plantenga MS Westerterp KR Under eating and underrecording of habitual food intake in obese men: selective underreporting of fat intake American Journal of Clinical Nutrition 2000 71 1 130 134 10617957 10.1093/ajcn/71.1.130

